# Archeological Tourist Destination Image Formation: Influence of Information Sources on the Cognitive, Affective and Unique Image

**DOI:** 10.3389/fpsyg.2019.02382

**Published:** 2019-10-22

**Authors:** Nuria Huete-Alcocer, Maria Pilar Martinez-Ruiz, Víctor Raúl López-Ruiz, Alicia Izquiedo-Yusta

**Affiliations:** ^1^Department of Spanish and International Economics, Econometrics, History and Economic Institutions, University of Castilla-La Mancha, Ciudad Real, Spain; ^2^Department of Economy and Business Administration, University of Castilla-La Mancha, Ciudad Real, Spain; ^3^Department of Economy and Business Administration, University of Burgos, Burgos, Spain

**Keywords:** information sources, tourism destination image, cognitive image, affective image, unique image

## Abstract

A destination’s image is a critical factor in tourists’ perceptions and evaluations of said destination. This paper analyzes the formation of the tourist destination image of Segóbriga Archeological Park, a cultural destination located in the province of Cuenca (Spain) that holds great heritage value. To this end, the paper adopted a multidimensional approach and used PLS-SEM to analyze the destination image, taking into account not only its cognitive and affective components, but also the unique image component. The latter has received less attention in the literature and is a novel factor among cultural destinations. The results show that this component is essential to the overall image of an archeological destination, but is not influenced by information sources.

## Introduction

With international tourism on the rise, destinations are locked in an increasingly intense competition for people’s attention. In order to survive in today’s global market, it is necessary for destinations to offer and manage a positive, yet differentiated image (Qu et al., 2011). Indeed, destination image is a fundamental factor in travelers’ selection of and behavior toward a destination (Gunn, 1972; Gartner, 1994; Bigné et al., 2001; Millet, 2010; Carballo et al., 2015; Elliot and Papadopoulos, 2016). This image is a mental concept formed from a set of impressions drawn from numerous information sources (Beerli and Martín, 2004); it is traditionally delineated into the cognitive and affective image. The former is created in the minds of tourists and depends on the quality and quantity of available information. The latter, by contrast, comprises the characteristics of the destination itself (Baloglu and Brinberg, 1997), since the information about this image is not only obtained from different sources, but also depends on the characteristics of each individual (Stern and Krakover, 1993; Beerli and Martín, 2004). Tourists can also form a third image – known as the unique image – when their experience with a destination leads them to find it distinct from all other destinations (Qu et al., 2011).

In this regard, information sources serve as an important antecedent of destination image (i.e., in the formation of this image), since travelers’ perceptions of a destination can be influenced by the information they receive from different sources. The literature has widely studied the connection between the use of information sources and destination image formation (e.g., Li et al., 2009; Coromina and Camprubí, 2016; Draper, 2016). However, what truly matters is how consumers process the information, which can depend on the type of message they receive (Rodríguez-Molina et al., 2015).

In light of the above, the present study aims to develop and test a theoretical model of destination image formation that addresses the influence of information sources on the formation of a cultural destination’s overall image – and specifically its different components (i.e., the cognitive, affective image, and unique images). Our aim is to study these image types more exhaustively and determine their role in the formation of a destination’s overall image. At the same time, this conceptual model seeks to fill a gap in the literature regarding the unique image – namely, what role it plays in the overall image and how it is affected by information sources. Researchers have not previously considered the unique image dimension in relation to the image formation process for a cultural destination such as an archeological site. We collected our data at the Segóbriga Archeological Park in the province of Cuenca (Spain), an archeological heritage destination that is regarded as one of the best-preserved Roman cities in the Western Roman Empire. Park visitors can first take a tour of all the best-preserved structures at the heritage site (walls, thermal baths, theater, amphitheater, basilica, circus, forum, necropolis, etc.). They can then supplement the tour with a visit to the interpretation center, which offers tourists an idea of what the ancient city was like. The site was granted the status of archeological park in order to promote tourism in the area and, by extension, boost the local economy.

## Conceptual Framework

### Destination Image

The concept of destination image has been extensively researched in tourism studies (Stepchenkova and Mills, 2010; Stepchenkova and Li, 2012; Cherifi et al., 2014; Sun et al., 2015; Fu et al., 2016), as well as in other disciplines such as sociology, environmental management and psychology (Tang, 2014), and marketing and consumer behavior (Sirakaya and Woodside, 2005; Stepchenkova and Morrison, 2008). There has also been a growing body of research on tourist destinations themselves (Gallarza et al., 2002), much of which builds on the work of Hunt, (1971); (Witter, 1985; Gartnerand and Hunt, 1987; Embacher and Buttle, 1989; Reilly, 1990; Echtner and Ritchie, 1991; Fakeye and Crompton, 1991). These myriad studies speak to the fact that image is fundamental to tourism destination promotion (Hudson et al., 2011), since the ways a destination differentiates itself is key to its success (Cai, 2002; Qu et al., 2011; Carballo et al., 2015).

The last four decades have produced a large body of research that illuminates the magnitude or significance of a destination’s image from different perspectives (Deng and Li, 2014). Destination image has been approached in terms of its dimension and conceptualization (Hunt, 1971, 1975; Gunn, 1972; Crompton, 1979; Gartner, 1989; Echtner and Ritchie, 1991; Baloglu and McCleary, 1999; Cai, 2002; Tasci et al., 2007; Tasci and Gartner, 2007; Stepchenkova and Morrison, 2008; Lai and Li, 2012, 2016), its evaluation and measurement (Gartner, 1989; Echtner and Ritchie, 1991, 1993, 2003; Fakeye and Crompton, 1991; Baloglu, 1998; Baloglu and McCleary, 1999; Beerli and Martín, 2004; Chen and Tsai, 2007; Stepchenkova and Morrison, 2008; Yang et al., 2012), changes in the image over time (e.g., Gartnerand and Hunt, 1987; Ahmed, 1991; Fakeye and Crompton, 1991; Kim and Morrsion, 2005), its management (e.g., Goodrich, 1978; Gartner, 1989; Baloglu, 1998; Konecnik and Gartner, 2007; Pike, 2009), its effect on tourists’ behavior (Chaudhary, 2000; Sirakaya and Woodside, 2005; Tasci and Gartner, 2007), and its formation (Gartner, 1989; Chon, 1990; Echtner and Ritchie, 1991; Beerli and Martín, 2004; Royo-Vela, 2009). In all these approaches, the importance of image in a destination’s tourism development is paramount (Martín-Santana et al., 2017). However, destination image formation is probably the most important of these issues (Deng and Li, 2014), as it underlies all the others (Gallarza et al., 2002).

One of the first studies on image formation was conducted by Gunn (1972), which looked at only two dimensions of the formation process: the organic image and the induced image. The organic image is understood as that arising from non-commercial or uncontrolled information sources, such as the opinions of friends, magazines, newspapers, news, reports, etc. (i.e., sources not intended to promote the destination). In contrast, the induced image arises from commercial information sources (i.e., marketing efforts of various commercial agents to publicize a destination, such as travel brochures; Tasci and Gartner, 2007). According to Gunn (1972, 1988), an individual’s image of a destination is constantly being reformed and renewed.

As a destination’s tourism image is essential to attracting tourists, it is important that said image be as real and current as possible. Indeed, the destination image clearly influences tourists’ behavior (Deng and Li, 2014), as it directly reflects visitors’ perceived quality of a place, as well as their satisfaction (evaluation of the stay) with, intention to return to (future behavior) and recommend (attitudinal loyalty) the destination (Bigné et al., 2001).

#### Components of the Destination Image

Each tourist’s individual perceptions can produce a relative and personal image a destination (Bigné et al., 2001; Gallarza et al., 2002; San Martín and del Bosque, 2008). In other words, tourists’ opinions are highly subjective, formed around very different antecedents based on their individual thoughts and emotions. As a result, many authors have generally investigated tourism destination image on the basis of its cognitive and affective components. For tourists, the cognitive image represents their knowledge of and beliefs regarding a place, while the affective image refers to their feelings or emotional responses toward it (Gartner, 1994; Baloglu, 1998; Beerli and Martín, 2004; Pike and Ryan, 2004; Royo-Vela, 2009; San Martín and del Bosque, 2008, 2011; Maher and Carter, 2011; Smith et al., 2015). The combination of the affective and cognitive images gives rise to the overall image (Baloglu and McCleary, 1999). Notably, the overall image reflects not only the common or shared aspects, but also the unique images that render a place distinctive (Echtner and Ritchie, 2003). The unique characteristics of a destination include both tangible attributes (e.g., beaches or historical sites) and intangible ones (e.g., customs, culture, and history) (Qu et al., 2011). Therefore, the unique image helps to sharpen destinations’ identities and thereby improve their competitive positioning (Lin and Kuo, 2018). However, few authors have examined this unique image component (e.g., Qu et al., 2011; Llodrà Riera, 2013; Lin and Kuo, 2018). The most important research on the topic has looked at only the two main components, i.e., the cognitive and affective images (Baloglu and Brinberg, 1997; Baloglu and McCleary, 1999; Kim and Richardson, 2003; Beerli and Martín, 2004; Hsu et al., 2004; Pike and Ryan, 2004; San Martín and del Bosque, 2008, 2011; Gutiérrez and del Bosque, 2010; Smith et al., 2015; Tan and Wu, 2016; Molinillo et al., 2018).

The present study will account for all three images types, with a special emphasis on the unique image. In this way, we hope to supplement the scant number of authors who have considered this third component in their work on destination image formation (e.g., Echtner and Ritchie, 1991; Qu et al., 2011; Llodrà Riera, 2013; Lin and Kuo, 2018). Below, we will briefly outline the research on all three image types before proceeding to discuss the role of each of them in the general image of Segóbriga.

(a) Cognitive image: Most studies have specifically considered the cognitive component of the image (Echtner and Ritchie, 1991; Echtner and Ritchie, 2003; Beerli and Martín, 2004; Chi and Qu, 2008; Sun et al., 2013). In fact, Pike (2002) found that of 142 papers published between 1973 and 2000, only six included the affective (or psychological) component. As noted, this dimension of the image refers to tourists’ knowledge or beliefs about the destination’s attributes (Baloglu and McCleary, 1999). The literature shows that this dimension arises from other elements, such as the natural environment, cultural resources, infrastructure, and quality. However, scholars differ on the importance they assign to these latter four factors (Baloglu and McCleary, 1999; Gallarza et al., 2002; Beerli and Martín, 2004; Chi and Qu, 2008; San Martín and del Bosque, 2008; Stylos et al., 2016; Stylidis et al., 2017). Some authors argue that these cultural and natural resources include particularities such as landscape beauty, cultural activities, or traditions and customs (e.g., Beerli and Martín, 2004; Stylos et al., 2016; Stylidis et al., 2017). Others maintain that these resources merely refer to attractions (e.g., Baloglu and McCleary, 1999).

(b) Affective image: To measure the affective image, many authors (e.g., Baloglu and McCleary, 1999; Bigné et al., 2001; Kim and Richardson, 2003; Beerli and Martín, 2004; Pike and Ryan, 2004; Chi and Qu, 2008; Nadeau et al., 2008; Alcañiz et al., 2009; Chen et al., 2016), have drawn on the work of Russell and Pratt (1980). Although the literature has spent less time addressing the affective component, it is particularly important for improving tourists’ perception of the destination image (Moreno-Gil et al., 2012), as it reflects their feelings toward a destination (Chen et al., 2016). In line with feelings-as-information theory, which explains how individuals make use of their emotions and impressions in ways that predispose them toward a destination (Kock et al., 2016). Stylos et al. (2016) found that advertisements featuring emotional content (e.g., words such as exciting, pleasant and relaxing) convey a destination’s affective image.

(c) Unique image: Considering that the tourism industry is marked by high competition and a relatively undifferentiated supply, Qu et al. (2011) found that it is necessary to identify the attributes that define a destination and make it unique in tourists’ minds. Other authors (e.g., Lin and Kuo, 2018) have only considered this component in order to study the overall image. Specifically, they concluded that the unique image is important for marketing differentiation strategies and, by extension, improving a place’s competitiveness. Thus, it appears that the unique image partly explains the influence of the affective image, meaning that the latter exerts a weaker effect on the overall image compared to the other two dimensions (cognitive and unique) (Qu et al., 2011). Hence, in the midst of increasing competition among tourist destinations, it is important to identify the attributes that characterize a cultural destination as unique and encourage tourists to perceive it as such.

### Information Sources in Image Formation

In reviewing the literature on destination image, it becomes clear that information sources are important antecedents to all three image components. Some researchers have suggested that these sources influence the formation of the cognitive image, but not the affective one (Woodside and Lysonski, 1989; Gartner, 1994; Baloglu and McCleary, 1999). In other words, external sources more strongly affect cognitive beliefs regarding a destination (Um and Crompton, 1990). Likewise, Beerli and Martín (2004) found that both organic sources (family members and friends) and autonomous sources (travel guides, news, articles, reports or documentaries) influence certain cognitive factors of the positive image. However, other authors (e.g., Jeong et al., 2012) have found that the information route is one of the primary factors influencing the cognitive image or overall image of a destination.

In recent years, academics and professionals have come to realize the importance of the Internet in shaping destination image. The Internet has transformed traditional WOM into eWOM. The communication of opinions is no longer done interpersonally (i.e., person-to-person or face-to-face), but rather is mediated by ICT (Huete-Alcocer, 2017). Although previous studies have shown that the Internet influences both the cognitive and affective dimensions of the destination image, most of the literature has focused solely on the cognitive dimension (Alcázar and Sicilia, 2015). In this regard, it is important to note that constructing a tourist destination image online is a more dynamic social process than traditional methods of image projection (e.g., through printed brochures and guides). The online image is instead generated by other tourists posting photographs, comments, perceptions, and experiences related to the destination (Hunter, 2016). For example, Kim et al. (2017) found that social media are a source of emerging information in tourism destination marketing. However, the issue of tourism destination image formation on social media remains relatively unexplored, especially empirically (Kim et al., 2017). Some authors (e.g., Prats et al., 2016) have suggested that an official website positively influences cognitive evaluations, since the information offered through such sites must be provided in a practical way to enable preparation of the trip. By contrast, guides influence affective evaluations due to appealing mainly to tourists’ emotions. Similarly, Martín-Santana et al. (2017) showed that the cognitive image is positively affected by high levels of online participation among tourists. Other authors (e.g., Molinillo et al., 2018) have found that tourist participation in online platforms positively impacts both the cognitive and affective images, and by extension, the intention to visit. Of course, image formation and the intention to visit vary according to the platform that travelers use to access the information (Molinillo et al., 2018). In general, most of the research agrees that the cognitive image somewhat informs the affective aspect, but the latter is also more difficult to change through external information (Li et al., 2009).

To our knowledge, no previous literature has sought to evaluate how information sources influence the unique image – and specifically that of an archeological destination. The present study aims to fill this gap, while also incorporating the cognitive and affective components.

### Proposed Model and Hypotheses

**FIGURE 1:**
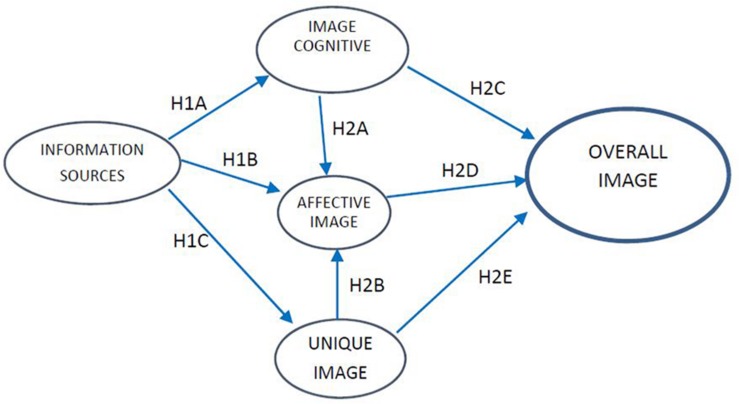
Model for the formation of the image of a cultural destination.

Drawing on the literature review, this study analyzed the image of a particular cultural destination as a reflective, multidimensional concept (Jarvis et al., 2003). The proposed model was developed based on previous studies showing that a destination’s image is fundamentally influenced by the cognitive and affective components, which are themselves influenced by the information sources that tourists utilize. The present study also analyzed the influence of the unique image component, in relation to both information sources and the overall image of an archeological heritage destination. We also considered the degree to which the cognitive and unique images influence the affective image. To this end, we propose the following conceptual model (Figure 1) and hypotheses:

Hypotheses H1 and H2 were formulated by drawing on Beerli and Martín (2004), Qu et al. (2011), and Llodrà Riera (2013). Hypothesis H1 aims to verify the influence of information sources on the formation of the different images: cognitive, affective, and unique. Its first source is the model proposed in Beerli and Martín (2004), which examined the role of information sources in the formation of cognitive and affective images. It second source is Qu et al. (2011), which included the unique image. Finally, it incorporates the study by Llodrà Riera (2013), which is one of the few other studies (outside of Echtner and Ritchie, 1991; Qu et al., 2011) to consider the unique image. Llodrà Riera (2013) also examined the role of information sources in the formation of the three types of images; however, that study referred to a sun-and-sand destination. In light of these considerations, we propose the following hypothesis alongside three sub-hypotheses (H1A, H1B, and H1C):

Hypothesis 1: The information sources used by tourists have a positive and significant influence on the cognitive, affective, and unique image.

H1A: Information sources positively and significantly influence the cognitive image.

H1B: Information sources positively and significantly influence the affective image.

H1C: Information sources positively and significantly influence the unique image.

In keeping with the above considerations and our objective (i.e., to analyze the formation of a cultural destination image), we relied on previous research (Beerli and Martín, 2004; Qu et al., 2011; Llodrà Riera, 2013) to formulate the second hypothesis (H2), which consists of five sub-hypotheses (H2A, H2B, H2C, H2D, and H2E). The aim of these sub-hypotheses was to analyze, first, how the cognitive image and unique image affect the affective image, and second, how each of these images (cognitive, affective, and unique) influences the destination’s overall image.

Hypothesis 2: With regard to the positive and significant influence of the cognitive and unique image on the affective image of a destination, and to the positive and significant influence of each of these images (cognitive, affective, and unique) on the overall image of a destination:

H2A: the cognitive image positively and significantly influences the affective image.

H2B: the unique image positively and significantly influences the affective image.

H2C: the cognitive image positively and significantly influences the overall image.

H2D: the affective image positively and significantly influences the overall image.

H2E: the unique image positively and significantly influences the overall image.

## Methodology

Of the many procedures used by various authors to analyze tourism destination images, we chose to use structural equation modeling (SEM) for this study. To this end, we found the PLS method to be the most efficient means of executing the SEM to test the proposed hypotheses.

### Data Collection Procedure

We first designed a questionnaire for the purpose of collecting information from site visitors following their visit. We devised the questions based on a thorough review of the literature and the specific characteristics of the destination under study. We conducted a pilot test with the questionnaire in order to ensure the tool’s clarity and relevance; only a few minor changes were made based on the results. The finalized survey was then administered to site visitors over a period of four consecutive weekends during the months of April and May 2017. A total of 598 questionnaires were given out to tourists aged 18 and over, resulting in a final sample of 511 valid responses.

A researcher handed out the questionnaires to the participants, gathered their consent, informed them about the study and procedure, and explained the meaning of some items. Participants were assured that their responses would remain anonymous and could be withdrawn from the study whenever they wanted. The authors will make the raw data available, without undue reservation, to any qualified researcher. With regard to ethics, the study was approved on March 15, 2017 by the Research Ethics Commission of the University of Castilla-La Mancha (chairperson: José Julian Garde López – Brea. Vice-chancellor’s office for Research and Scientific Policy; secretary: Isabel Turégano Mansilla, Cuenca Faculty of Social Sciences; members: Inés Martínez Galán, Christian Gortázar Schmidt and Jorge Laborda Fernández).

### Variables and Measurement Scales

Most of the constructs used in the present research are multidimensional concepts, each comprising several items. For all items, we employed a 5-point Likert scale as the measurement method. We analyzed a total of 32 items concerning information sources, including 19 about traditional sources and 13 about online ones (Tables 1, 2, respectively).

**TABLE 1:** Traditional information sources construct.

**Questionnaire question**	**Item**	**Acronym**
1. Indicate the extent to which you have used the following traditional information sources to obtain information about Segóbriga Archeological Park. SCALE: 1 = I did not use it at all. 5 = It was one of my main sources of information. **Authors:** Gunn, 1988; Gartner, 1994; Bigné et al., 2001; Beerli and Martín, 2004; Llodrà Riera, 2013	Tourist brochures	TSOURCE1
	Travel agencies or tour operator tour	TSOURCE2
	Public figures with a recognized audience	TSOURCE3
	Scientific papers on Segóbriga	TSOURCE4
	News, reports and documentaries	TSOURCE5
	Tourist information from agencies that promote the destination (e.g., ADESIMAN, Ministry of Education, Culture and Sports, Ministry of Tourism, Trade and Crafts)	TSOURCE6
	Schools (primary schools, universities, vocational schools)	TSOURCE7
	Nearby accommodations, supplementary offer	TSOURCE8
	Specialized tourism media	TSOURCE9
	Specialized archeological heritage media	TSOURCE10
	Books	TSOURCE11
	Travel guides	TSOURCE12
	Fairs	TSOURCE13
	TV shows and movies	TSOURCE14
	Radio	TSOURCE15
	The Internet	TSOURCE16
	Friends and family	TSOURCE17
	Local residents	TSOURCE18
	The sign located on the A-3 highway: Madrid – Valencia – Alicante.	TSOURCE19

**TABLE 2:** Online information sources construct.

**Questionnaire question**	**Item**	**Acronym**
2. Indicate the extent to which you used the following online information sources to obtain information about Segóbriga Archeological Park. SCALE: 1 = I did not use it at all. 5 = It was one of my main sources of information **Authors:** Llodrà Riera, 2013; Zeng and Gerritsen, 2014; Llodrà-Riera et al., 2015; Tseng et al., 2015; Coromina and Camprubí, 2016	Official website of the site (www.segóbriga.org)	ONSOURCE1
	Official website of the site (www.spaincenter.org)	ONSOURCE2
	Social media (Facebook, Twitter, Instagram, etc.)	ONSOURCE3
	Blogs	ONSOURCE4
	Websites with user ratings (TripAdvisor)	ONSOURCE5
	Websites of tourism companies in Cuenca that offer it	ONSOURCE6
	Website of the Provincial Government of Cuenca	ONSOURCE7
	Website of the Regional Government of Castilla-La Mancha (www.patrimoniohistoricoclm.es)	ONSOURCE8
	Official Castilla-La Mancha tourism website (www.turismocastillalamancha.es)	ONSOURCE9
	Internet search engines (Google, Bing, Yahoo, etc.)	ONSOURCE10
	Maps (Google Maps, ViaMichelín, Guía Repsol, etc.)	ONSOURCE11
	Forums (Los Viajeros, TripAdvisor)	ONSOURCE12
	Video-sharing apps (YouTube)	ONSOURCE13

When carrying out the CFA for the information sources (traditional and online), only seven factors emerged, which grouped several items eliminating those whose value of lambda was less than 0.5: SOURINF1 (scientific articles of Segóbriga, news, reports and documentaries and books), SOURINF2 (media specialized in tourism, media specialized in archeological heritage, travel guides, fairs and TV series and movies), SOURINF3 (only WOM), SOURINF4 (promotion agencies, Web of the Diputación of Cuenca, Web JCCM and Web tourism of Castilla-La Mancha), SOURINF5 (Webs with user ratings and forums) SOURINF6 (blogs and Webs of tourism business Cuenca) and SOURINF7 (Internet browser and Google Maps).

Table 3 shows the variables used to measure the cognitive dimension, which were based on Beerli and Martín (2004) and Qu et al. (2011). While numerous studies clearly signal that this dimension comprises the natural environment, cultural resources, infrastructure, and quality, the importance assigned to each varies (e.g., Baloglu and McCleary, 1999; Gallarza et al., 2002; Beerli and Martín, 2004; Chi and Qu, 2008; San Martín and del Bosque, 2008). For example, while some authors (e.g., Beerli and Martín, 2004) suggest that cultural resources are features of landscape beauty, traditions, customs or cultural activities themselves, others (e.g., Baloglu and McCleary, 1999) consider such resources to be mere attractions.

**TABLE 3:** Cognitive image construct.

**Questionnaire question**	**Variable**	**Item**	**Acronym**
3. Rate the quality of each of these elements: SCALE: 1 = Most negative score 5 = Most positive score **Authors:** Beerli and Martín, 2004; Qu et al., 2011; Stylos et al., 2016; Stylidis et al., 2017	Natural resources	Weather	COGIMA1
		Richness of the landscape	COGIMA2
	General infrastructure	Ability to access the archeological park by public transport	COGIMA3
		Ability to access the archeological park by private transport	COGIMA4
	Tourism infrastructure	Accessibility inside the site	COGIMA5
		Restaurants	COGIMA6
		Hotels and accommodations	COGIMA7
		Ease of obtaining tourist information at the archeological park	COGIMA8
	Leisure and recreation tourism	Activities within the archeological park (educational and leisure activities, sports events such as races, photography contests, exhibitions)	COGIMA9
	Culture, history and art	Monuments	COGIMA10
		Museum and pieces	COGIMA11
		Historical constructions	COGIMA12
		Customs and ways of life	COGIMA13
		Food	COGIMA14
		Theater festivals	COGIMA15
		Concerts	COGIMA16
		Crafts	COGIMA17
		Folklore	COGIMA18
		Guided tours of the archeological park	COGIMA19
	Natural environment	Maintenance and conservation	COGIMA20
		Cleaning	COGIMA21
		Security service at the archeological park	COGIMA22
		Attractiveness of the site	COGIMA23
	Social environment	Hospitality and friendliness of local residents	COGIMA24
		Local quality of life	COGIMA25
	Political and economic factors	Value for money of the admission to the archeological park	COGIMA26

On the other hand, the CFA for the cognitive image only revealed six factors after we eliminated items whose lambda value was less than 0.5: COGNITIVE1 corresponds to the measured variable of natural resources (climate and richness of the landscape); COGNITIVE2 with tourist infrastructures (restaurants, hotels and accommodation); COGNITIVE3 with elements of the culture, history and art (monuments, museum and pieces, historical constructions, customs and ways of life); COGNITIVE4 includes another five items of the variable culture, history and art (gastronomy, theater festivals, concerts, crafts and folklore); COGNITIVE5 corresponds to the variable natural environment, measured by the maintenance and conservation of the deposit; and finally, COGNITIVE6 reflects the social environment (hospitality and friendliness of local residents and quality of life in the area).

We drew on several studies, most of them built on the findings of Russel and Pratt, to measure the affective image (e.g., Baloglu and McCleary, 1999; Bigné et al., 2001; Kim and Richardson, 2003; Beerli and Martín, 2004; Pike and Ryan, 2004; Chi and Qu, 2008; Nadeau et al., 2008; Alcañiz et al., 2009; Chen et al., 2016; Stylos et al., 2016; Stylidis et al., 2017). These studies, in turn, built on the findings of Russell and Pratt (1980). Table 4 shows the attributes that we considered.

**TABLE 4:** Affective image construct.

**Questionnaire question**	**Authors**	**Item**	**Acronym**
4. Indicate the degree to which you agree or disagree with the following statements. Segóbriga Archeological Park is: SCALE: 1 = Strongly disagree 5 = Strongly agree	Hosany et al., 2007; Qu et al., 2011	Beautiful	AFFIMA1
		Ugly	AFFIMA2
	Baloglu and McCleary, 1999; Bigné et al., 2001; Kim and Richardson, 2003; Beerli and Martín, 2004; Pike and Ryan, 2004; Chi and Qu, 2008; Nadeau et al., 2008; Alcañiz et al., 2009; Chen et al., 2016; Stylos et al., 2016; Stylidis et al., 2017	Nice	AFFIMA3
		Unpleasant	AFFIMA4
		Relaxing	AFFIMA5
		Stressful	AFFIMA6
		Fun	AFFIMA7
		Boring	AFFIMA8
		Exciting	AFFIMA9
		Depressing	AFFIMA10

With regard to measuring the unique image dimension, we drew on three previous studies: namely, Echtner and Ritchie (1991), Qu et al. (2011), and Llodrà Riera (2013) (Table 5). This variable captures the characteristics of the site that visitors regard as unique relative to other archeological parks. The associated CFA revealed only three factors for the unique image (all except UNIMA 1).

**TABLE 5:** Unique image construct.

**Questionnaire question**	**Authors**	**Item**	**Acronym**
5. Would you say that Segóbriga Archeological Park offers a unique experience in the following cases? SCALE: 1 = Strongly disagree 5 = Strongly agree	Echtner and Ritchie, 1991; Qu et al., 2011; Llodrà Riera, 2013	When sports activities are held there.	UNIMA1
		When cultural activities are held there (e.g., conferences, plays, etc.)	UNIMA2
6. Do you agree that the visit to the archeological park’s interpretation center offered a unique experience? SCALE: 1 = Strongly disagree 5 = Strongly agree			UNIMA3
7. Do you agree that the visit to Segóbriga Archeological Park was a unique experience compared to other parks? SCALE: 1 = Strongly disagree 5 = Strongly agree			UNIMA4

The CFA of the affective image revealed only three factors: AFFIMA1, AFFIMA3, and AFFIMA5 (Table 6).

**TABLE 6:** Measurement instrument: individual reliability.

**Factor**	**Indicator**	**Loading**
INFORMATION SOURCES	SOURINF1	0.998
	SOURINF2	0.998
	SOURINF3	0.708
	SOURINF4	0.997
	SOURINF5	0.996
	SOURINF6	0.999
	SOURINF7	0.953
COGNITIVE IMAGE	COGNITIVE1	0.889
	COGNITIVE2	0.960
	COGNITIVE3	0.977
	COGNITIVE4	0.964
	COGNITIVE5	0.972
	COGNITIVE6	0.880
AFFECTIVE IMAGE	AFFIMA1	0.868
	AFFIMA3	0.880
	AFFIMA5	0.744
UNIQUE IMAGE	UNIMA2	0.701
	UNIMA3	0.873
	UNIMA4	0.855
OVERALL IMAGE	N/A	N/A

*N/A, Not applicable to these variables as they were measured with a single item and thus would have a value of 1.*

Finally, we measured the site’s overall image using a single question based on Baloglu and McCleary (1999): “After your visit, rate the site’s overall image.” Respondents had to score the question on a 5-point Likert scale (1 = Very bad/5 = Very good).

The survey concluded with a final set of questions related to respondents’ demographics, such as age, income level, gender, education, overnight stay in the area, place of origin, etc. Based on this data, we verified the profile of the typical tourist to Segóbriga: a middle-aged (between 40 and 59 years old) person, most often a woman, who works for someone else, has a higher education, and earns a rather high income.

## Analysis of Results

### Evaluation of the Measurement Model: Validity and Reliability

Before testing the proposed hypotheses, we evaluated the measurement model with PLS (Barclay et al., 1995). Specifically, we employed SmartPLS 3.0 software to analyze the individual reliability of each item, the reliability of the scale, and the convergent and discriminant validity. We obtained the significance of the parameters through bootstrapping, which assesses the accuracy of the PLS estimates (Hair et al., 2011).

#### The Individual Reliability of Each Item

The individual reliability of each indicator was calculated, and the simple correlations of the means with their constructs were analyzed. Those items with a factor loading greater than or equal to 0.707 (meaning that more than 50% of the observed variable’s variance was shared with the construct) were considered reliable (Carmines and Zeller, 1979).

#### Composite Reliability

Next, we calculated the composite reliability using Cronbach’s alpha (Cronbach, 1951), which assesses whether the indicators measure the construct to which they are assigned. Table 7 shows that all the constructs met the threshold for reliability (i.e., a Cronbach’s alpha value equal to or greater than 0.7).

**TABLE 7:** Measurement instrument: composite reliability.

**Factor**	**Cronbach’s alpha**	**Composite Reliability**
Information sources	0.983	0.986
Cognitive image	0.974	0.979
Affective image	0.780	0.871
Unique image	0.733	0.849
Overall image	1.000	1.000

#### Convergent and Discriminant Validity

First, we assessed the convergent validity by calculating the average variance extracted (AVE) for each construct (Fornell and Larcker, 1981). According to Fornell and Larcker (1981), this measure reflects a construct’s amount of variance due to its indicators versus mere measurement error. Its value should be equal to or greater than 0.5, which indicates that each construct explains at least 50% of the assigned indicators’ variance. We also calculated rho_A, which is another of the most important reliability measures for PLS (Dijkstra and Henseler, 2015; Table 8).

**TABLE 8:** Measurement instrument: convergent validity.

**Factor**	**AVE**	**Rho_A**
Information sources	0.912	1.000
Cognitive image	0.886	0.992
Affective image	0.694	0.810
Unique image	0.654	0.764
Overall image	1.000	1.000

Subsequently, we checked the discriminant validity, which captures the extent to which a given construct is different from the others in the model. This validity requires that the variance shared by a variable and its indicators must be greater than the variance shared with the model’s other variables (Barclay et al., 1995). There are two evaluation methods: an analysis of the cross-loadings or through the correlations of the latent variables (AVE). The present research used the latter method, as can be seen in Table 9, which shows the data from the correlation matrix between the model’s constructs (Table 9). The diagonal of the matrix shows the value of the square root of the AVE of the corresponding construct (bolded values in Table 9). As can be seen, the correlations between the constructs were less than the square root of the AVE. Therefore the constructs met the requirement for discriminant validity. Additionally, to check that measuring instrument, we also calculated the HTMT values: As Table 9 evidences, the values were consistently less than 0.9 (Gold et al., 2001).

**TABLE 9:** Measurement instrument: discriminant validity (Fornell-Larcker criterion).

	**Information sources**	**Cognitive image**	**Affective image**	**Unique image**	**Overall image**
Information sources	**0.955**				
Cognitive image	0.073 (0.055)	**0.941**			
Affective image	−0.072 (0.079)	0.359 (0.415)	**0.833**		
Unique image	0.029 (0.055)	0.339 (0.351)	0.403 (0.477)	**0.809**	
Overall image	−0.048 (0.044)	0.337 (0.357)	0.525 (0.582)	0.570 (0.650)	**1.000**

*Bolded values on the diagonal are the square root of the AVE of the corresponding construction.*

### Evaluation of the Structural Model

Table 10 displays the results of the structural analysis carried out with PLS. The path coefficients (β) indicate the relationships between the structures, as well as the significance of these relationships. In order to study the stability and significance of the estimated parameters, we applied the aforementioned non-parametric resampling technique: bootstrapping. This technique involves creating a number of bootstrap samples through a randomized repeated sampling of the original sample (Hair et al., 2011).

**TABLE 10:** Structural analysis of the hypothesis tests.

**Hypothesis**	**Structural relationship**	**Standardized path (β)**	**Bootstrap *t*-value**	**Support for hypothesis**
H1A	Information sources→Cognitive image	0.073	5,293	SUPPORTED
H1B	Information sources →Affective image	-0.100	5,255	SUPPORTED (-)
H1C	Information sources→Unique image	0.029	1,493	NOT SUPPORTED
H2A	Cognitive image→Affective image	0.259	19,537	SUPPORTTED
H2B	Unique image → Affective image	0.318	20,731	SUPPORTED
H2C	Cognitive image →Overall image	0.080	5,729	SUPPORTED
H2D	Affective image →Overall image	0.331	22,641	SUPPORTED
H2E	Unique image →Overall image	0.409	27,841	SUPPORTED

As Table 10 indicates, all the accepted direct effects held true at a significance level of 99%. These results allowed us to draw the following conclusions: First, we found support for sub-hypothesis H1A, which sought to verify whether information sources had a positive and significant influence on the cognitive image (β = 0.073, *p* < 0.001). However, the support for sub-hypothesis H1B – which sought to confirm the influence and significance of information sources with regard to the affective image – diverged from our expectations (β = –0.1, *p* < 0.001). As can be seen, the data were significant, but the sign of the coefficient was negative. In other words, the influence of one variable on the other was negative. This may be because the relationship between the two variables is very weak or is being impacted by a mediating variable. Finally, we found no support for sub-hypothesis H1C, which sought to test the positive and significant influence of information sources on the unique image (β = 0.029, *p* > 0.001, *p* > 0.01, *p* > 0.05). Specifically, the coefficient was not significantly different from zero, since the empirical value of t was less than the critical value of t for significance levels of 0.90, 0.95, and 0.99.

With regard to the second hypothesis (H2), we found support for all five sub-hypotheses (H2A, H2B, H2C, H2D, and H2E). Thus, we confirmed that the cognitive image has a positive and significant influence on the affective image (H2A) (β = 0.259, *p* < 0.001), as does the unique image (H2B) (β = 0.318, *p* < 0.001). Meanwhile, the cognitive (H2C) (β = 0.080; *p* < 0.001), affective (H2D) (β = 0.331; *p* < 0.001), and unique image (H2E) (β = 0.409; *p* < 0.001) all exerted a significant effect on the overall image.

In order to assess the structural model, we calculated R2 (Table 11). According to Falk and Miller (1992), the explained variance of the endogenous variables (R2) should be equal to or greater than 0.1. However, an increasingly common alternative to solely considering R2 is the predictive relevance criterion Q2, proposed by Chin (1998): 318: Q2 measures how well the studied values can be reconstructed by the model and its parameters. If Q2 is greater than zero, the model has predictive relevance; if it is less than or equal to zero, it does not. But this is a rule-of-thumb that does not take into account the sampling distribution of Q2 (Shmueli et al., 2016; Arias-Oliva et al., 2019). As shown in Table 11, the R2 values were greater than 0.1 for all the variables except the cognitive image and the unique image. Likewise, all the Q2 values were greater than zero. Therefore, we can confirm that the model has predictive relevance.

**TABLE 11:** Predictive relevance of the model.

**Factor**	***R*^2^**	***Q*^2^**
Information sources	0.000	0.000
Cognitive image	0.005	0.002
Affective image	0.229	0.145
Unique image	0.001	0.001
Overall image	0.434	0.410

**FIGURE 2:**
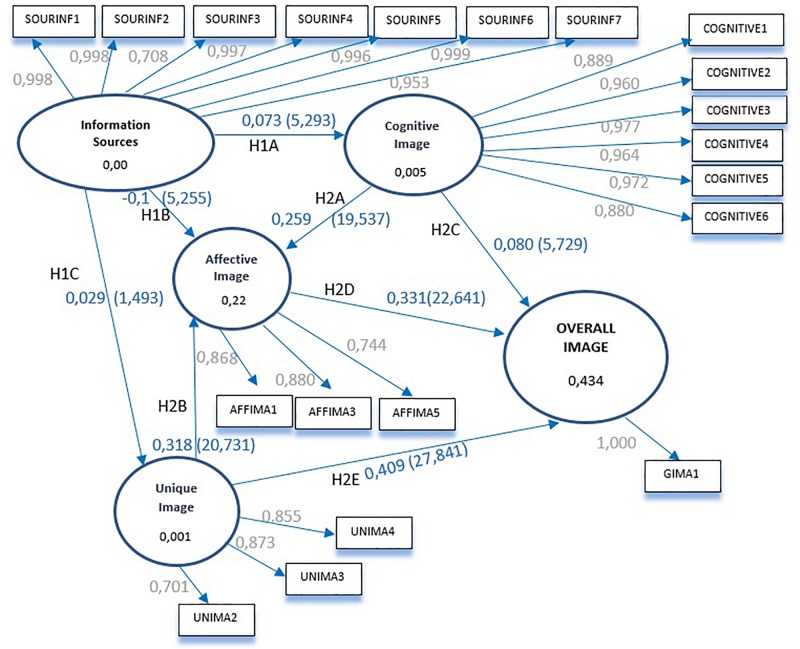
Result of the model SEM.

Finally, we calculated the value of the standardized root mean square residual (SRMR) (Henseler et al., 2015) in order to measure the model’s fit and compare the difference between the observed and predicted correlations. Values less than 0.08 are considered acceptable. Our proposed model achieved a value of 0.048 and thus had an appropriate fit. Figure 2 below illustrates the resulting SEM model.

## Discussion

Like other authors (e.g., Beerli and Martín, 2004; Llodrà Riera, 2013), we could only verify that information sources have a positive and significant influence on the cognitive image (sub-hypothesis H1A). Meanwhile, information sources had a significant negative impact on the affective image (H1B) and no significant impact on the unique image (H1C). These results are largely contrary to those obtained by Llodrà Riera (2013), who found support for all three sub-hypotheses.

In line with previous reports (e.g., Baloglu and McCleary, 1999; Beerli and Martín, 2004; Qu et al., 2011; Llodrà Riera, 2013), the results showed that cognitive image positively influences the overall image (H2C). Likewise, the influence of the cognitive image on the affective image (H2A) was consistent with previous findings (Baloglu and McCleary, 1999; Beerli and Martín, 2004). The fact that the affective image clearly influenced the overall image (H2D) confirms the findings of Baloglu and McCleary (1999), Beerli and Martín (2004), Ekinci and Hosany (2006), Qu et al. (2011), and Papadimitriou et al. (2015). Collectively, these findings suggest that the affective image can have a very significant direct effect on the overall image. Additionally, we found that the unique image influences both the affective (H2B) and overall image (H2E). Interestingly, its effect on the overall image was even greater than that of the affective image, although the latter has received considerably more attention in the literature. This is consistent with the findings of Qu et al. (2011), but not of Llodrà Riera (2013), the latter of whom was unable to confirm the relationships between the unique, affective, and overall images.

## Conclusion

This study sought to examine the image formation of a particular cultural and tourism destination, Segóbriga Archeological Park. Both the theoretical review of the concept and the accompanying quantitative studies revealed that the overall image of this destination is a multidimensional phenomenon consisting of cognitive, affective, and unique dimensions. In other words, tourists form perceptions of Segóbriga based on not only their individual knowledge and beliefs regarding the site’s characteristics, but also their feelings toward and perceptions of the destination as a unique experience. The results also illuminate that the unique image influenced the affective image more than the cognitive one. Additionally, the unique image had the greatest influence on the overall image (followed by affective and then cognitive). The fact that the unique image significantly influenced the overall image is consistent with the findings of Echtner and Ritchie (1993), who noted that the unique image is an excellent source of differentiation that can help improve the overall image.

Likewise, our study verified the role played by information sources in image formation. By analyzing the influence of these sources on the three dimensions of the image (cognitive, affective, and unique), we found that they positively and significantly influence the cognitive image, negatively influence the affective image, and do not seem to significantly influence the unique image.

The results also underscore the information sources most often used by tourists who visit this type of site: The top-ranking one was the Internet (an induced source), followed by word of mouth (WOM) from friends and family (an organic source). These sources thus carry the greatest weight in the destination image formation process. Consequently, tourism promotion and management would be best served by leveraging online sources (social media, websites, etc.) that allow users to post-comments (eWOM) that might be seen by potential future tourists. In short, eWOM is a powerful means of promoting tourism.

### Practical and Theoretical Implications

The above findings constitute a novel contribution to the literature. First, the present study highlights the importance of the affective image. This stands in contrast to most research to date, which has placed more emphasis on the cognitive image. Second, we provide a test of the unique image dimension, which has largely been ignored in the literature. To this end, we confirmed that the unique image is an essential component of the overall image for an archeological destination. These practical implications align with those of Qu et al. (2011), who argued that tourists form a unique image impression following their visits, which helps to distinguish a destination in their minds. Thus, it appears that the unique image can bolster marketing differentiation strategies and make a destination more competitive (Lin and Kuo, 2018). Consequently, academics should continue researching this image component in relation to other tourist destinations.

Moreover, destination managers should take into account that potential and actual tourists are increasingly using the Internet to find information or post-comments on social media. In our study, respondents assigned the highest scores to the destination’s website, to search engines (such as Google, Bing, or Yahoo), and to online map services (such as Google Maps, ViaMichelín, or Repsol Guide). Based on these results and the prior literature, we can safely conclude that information sources can be leveraged as promotional tools to positively influence a destination’s image formation.

In conclusion, this research analyzed the factors that influence tourists’ perceived images of an archeological park. In aiming to improve the future management of such sites, our results highlight ways of attracting potential tourists, encouraging recommendations, and increasing the intention to return.

### Limitations and Further Research

The present study features several limitations worth highlighting. For practical purposes, we measured the constructs from before and after the visit at the same time (i.e., following respondents’ visit to the site). Consequently, it was not possible to gauge respondents’ assessments of the site’s image prior to their tourism experience. Additionally, we discarded several constructs, and the relationships between them, when constructing the proposed conceptual model. Future research should explore some of these variables and relationships. For example, it would be useful to analyze what motivates tourists’ to finally decide to visit this destination and see how these factors influence each of the image components (cognitive, affective and unique). It may be that tourists develop different perceptions about this type of destination’s overall image based on their motivation (or lack thereof). Future studies should also explore how these socio-demographic characteristics (age, residence, work, level of studies, etc.) relate to people’s willingness to visit such archeological destinations.

Possibly, a limitation may be the cognitive effect might not influence of the overall image, due to the existence of an indirect effect through the affective image.

Other avenues for future research include assessing how tourists’ perceptions of a destination change before, during, and after the visit, as well as analyzing the role that local residents play in the formation of the different image components.

## Data Availability Statement

The raw data supporting the conclusions of this manuscript will be made available by the authors, without undue reservation, to any qualified researcher.

## Ethics Statement

The studies involving human participants were reviewed and approved by Research Ethics Commission of the University of Castilla-La Mancha. The patients/participants provided their written informed consent to participate in this study.

## Author Contributions

All authors listed have made a substantial, direct and intellectual contribution to the work, and approved it for publication.

## Conflict of Interest

The authors declare that the research was conducted in the absence of any commercial or financial relationships that could be construed as a potential conflict of interest.
